# Primeira Realização de Implante Transcateter de Valva Tricúspide Via Transjugular com o Sistema Lux-Valve Plus Na América Latina. Um Relato de Caso

**DOI:** 10.36660/abc.20240201

**Published:** 2024-10-08

**Authors:** Vinicius Esteves, Pedro Beraldo de Andrade, Sergio Kreimer, Fernanda Almeida Esteves, Francisco Monteiro de Almeida Magalhães, Thomas Modine

**Affiliations:** 1 Rede D'Or Sao Luiz São Paulo SP Brasil Rede D'Or Sao Luiz, São Paulo, SP – Brasil; 2 Santa Casa de Misericórdia de Marília Marília SP Brasil Santa Casa de Misericórdia de Marília – Cardiologia Invasiva, Marília, SP – Brasil; 3 Hospital e Maternidade Brasil Santo André SP Brasil Hospital e Maternidade Brasil, Santo André, SP – Brasil; 4 Hospital das Clínicas da Faculdade de Medicina da Universidade de São Paulo Instituto do Coração São Paulo SP Brasil Instituto do Coração do Hospital das Clínicas da Faculdade de Medicina da Universidade de São Paulo, São Paulo, SP – Brasil; 5 Centre Hospitalier Universitaire de Bordeaux Bordeaux França Centre Hospitalier Universitaire de Bordeaux, Bordeaux, Nouvelle-Aquitaine – França

**Keywords:** Insuficiência da Valva Tricúspide, Insuficiência Cardíaca, Implante de Prótese de Valva Cardíaca

## Introdução

A prevalência de regurgitação tricúspide (RT) aumenta com a idade, doença cardíaca esquerda concomitante e fibrilação atrial crônica.^1^ A RT moderada ou grave está associada a mortalidade excessiva e desfechos desfavoráveis.^2^ O tratamento de escolha é o reparo da valva tricúspide, com um anel protético capaz de reduzir o diâmetro do anel tricúspide, melhorar a coaptação dos folhetos valvares e corrigir a regurgitação. A troca valvar é reservada aos pacientes que não apresentam condições anatômicas para reparo. Entretanto, a abordagem cirúrgica isolada da valva tricúspide continua raramente indicada e ainda não há dados que demonstrem melhora da sobrevida apenas com o tratamento cirúrgico da RT, tornando esta condição subtratada na prática clínica, apesar do aumento da taxa de mortalidade em pacientes com RT moderada a grave conduzidos clinicamente.^3^

Com base nestas observações, recentemente o tratamento da RT vem transicionando de uma abordagem conservadora para uma abordagem mais intervencionista. Essa mudança levou às primeiras tentativas em humanos de intervenções transcateter na válvula tricúspide (ITVT). Vários dispositivos foram desenvolvidos, com estratégias baseadas na redução do anel valvar, na melhora da coaptação entre os folhetos ou na substituição valvar.^4^ Em um estudo com pareamento por escore de propensão, o ITVT foi associado a uma sobrevida 40% maior e à ausência de reinternação por insuficiência cardíaca.^5^

A LuX-Valve (Jenscare Biotechnology Co., Ningbo, China) é um dispositivo de substituição de válvula tricúspide transcateter (SVTT) ortotópico autoexpansível, cuja viabilidade e eficácia foram previamente relatadas.^6,7^ O sistema LuX-Valve Plus é a versão de segunda geração do dispositivo e pode ser implantado através da veia jugular.^8^ Apresentamos a primeira realização de SVTT transjugular com o sistema LuX-Valve Plus na América Latina em uma paciente com RT sintomática de alto risco cirúrgico.

## Relato de Caso

Paciente do sexo feminino, 78 anos, portadora de hipertensão arterial sistêmica, fibrilação atrial, diabetes mellitus, dislipidemia, doença arterial obstrutiva periférica e artrite reumatoide, apresentava sinais e sintomas de insuficiência cardíaca direita crônica, incluindo edema periférico, ascite, intolerância ao exercício, dispneia e baixa capacidade funcional. Apesar da terapia clínica otimizada, a paciente apresentou internações hospitalares recorrentes por piora dos sintomas. O ecocardiograma revelou função biventricular preservada, sem sinais de hipertensão arterial pulmonar, aumento biatrial significativo (volume indexado de átrio direito = 50mL/m^2^ e volume indexado de átrio esquerdo = 75mL/m^2^), RT grave (3+), com área de orifício regurgitante (ERO) efetivo = 0,4cm^2^, volume regurgitante = 42mL/batimento e diâmetro do anel tricúspide = 46mm. Com base na idade avançada, risco de mortalidade em 30 dias de 8,4% pelo escore da *Society of Thoracic Surgeons* (STS) e anatomia favorável pela análise da tomografia computadorizada, o *Heart Team* optou pela SVTT com sistema LuX-Valve Plus 30-50.

**Figura 1:**
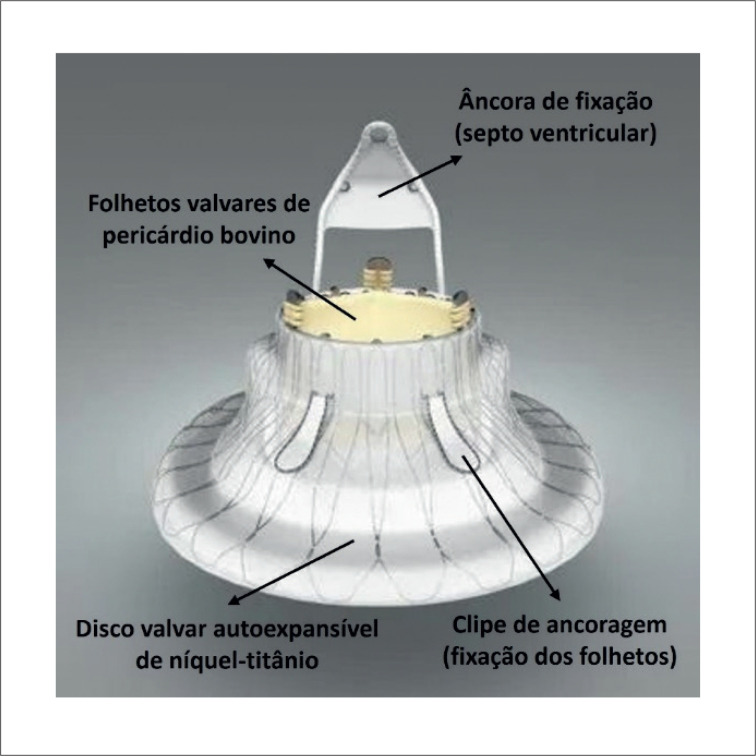
Sistema LuX-Valve Plus.

**Figura 2:**
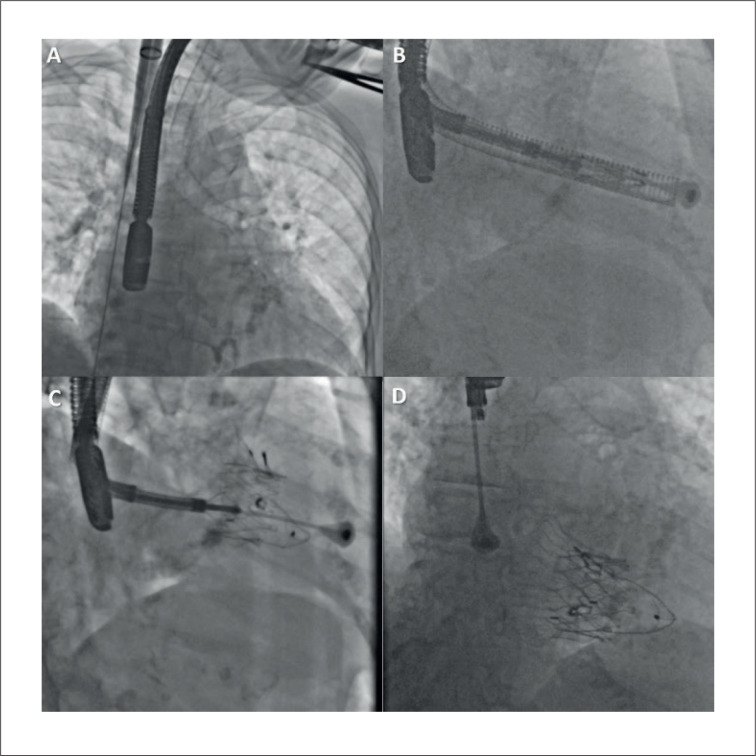
Técnica de implante do dispositivo LuX-Valve Plus. A) Acesso percutâneo transjugular. B) Alinhamento e coaxialização do sistema. C) Liberação ventricular. D) Liberação final.

O sistema LuX-Valve Plus é composto por quatro componentes: 1) válvula protética de três folhetos de pericárdio bovino; 2) Stent valvular de nitinol autoexpansível constituído por um disco atrial; 3) uma âncora ventricular septal, e 4) dois clipes de ancoragem revestidos com politetrafluoretileno (Figura 1). O procedimento foi realizado sob anestesia geral com orientação ecocardiográfica transesofágica e fluoroscópica. A veia jugular interna direita foi puncionada sob orientação ultrassonográfica, uma bainha introdutora de 30 Fr foi então posicionada na veia e, posteriormente, o sistema de liberação foi avançado. A válvula foi liberada e os clipes de preensão do folheto anterior foram expandidos. O sistema de entrega foi então retirado, permitindo a captura do folheto anterior pelos clipes. O disco atrial foi posteriormente implantado e a válvula começou a funcionar. O componente de ancoragem foi então posicionado e fixado pela âncora de nitinol de três pontas no septo ventricular. Por fim, a válvula foi completamente liberada e o cateter introdutor foi retirado (Figura 2). A hemostasia foi obtida com 2 Perclose ProGlide™.

O ecocardiograma na alta demonstrou redução significativa da RT, com refluxo menor que leve em controle de 30 dias. A paciente referiu melhora significativa da classe funcional pela *New York Heart Association* (NYHA) aos 30 dias de acompanhamento.

## Discussão

O sistema LuX-Valve Plus é um sistema de SVTT implantado pela veia jugular. O sistema de entrega tem as funções de flexão, rotação e expansão, o que permite atender aos requisitos de posicionamento da válvula e garantir que a válvula possa ser coaxializada com a válvula tricúspide nativa. Os resultados de doze meses do implante do sistema LuX-Valve de primeira geração em seis pacientes, através de uma pequena incisão no tórax direito e no átrio direito, confirmaram sua segurança e eficácia, com melhora significativa no gradiente transvalvar médio, na sobrecarga de câmaras direitas, nos índices de função ventricular e *strain* longitudinal global, na redução da classe funcional pela NYHA e ausência de vazamento paravalvar significativo em todos os pacientes, exceto um, que faleceu três meses após a intervenção devido a RT paravalvar moderada e não recuperação da insuficiência cardíaca direita.^7^

A primeira experiência em humanos com este dispositivo de segunda geração demonstrou sucesso do procedimento em todos os dez casos realizados, sem mortalidade intraprocedimento ou conversão para cirurgia aberta. Diferentemente do nosso caso, cujo acesso foi totalmente percutâneo, os procedimentos foram realizados através de corte de 3 a 4 cm na pele do lado direito do pescoço e exposição da veia jugular direita. Todos os pacientes relataram melhora da classe funcional e RT menor que leve no controle ecocardiográfico de 30 dias.^8^ Pacientes com hipertensão arterial pulmonar grave (pressão sistólica da artéria pulmonar ≥55 mmHg), outras lesões valvares que requeressem intervenção cirúrgica, fração de ejeção do ventrículo esquerdo <50%, anomalia congênita de Ebstein, displasia arritmogênica do ventrículo direito ou doença arterial coronariana grave não tratada foram excluídos.

A intervenção transcateter mais utilizada em pacientes com insuficiência cardíaca direita é o reparo transcateter tricúspide borda a borda. No entanto, muitos pacientes tornam-se inadequados para a técnica, pois apresentam grandes falhas de coaptação, *tethering* de folhetos e RT torrencial. Para estes pacientes, a SVTT surgiu como uma alternativa atrativa, apesar de um registro de mundo real sobre a seleção de candidatos a SVTT demonstrar uma elevada taxa de exclusão, principalmente relacionada com o grande diâmetro do anel tricúspide.^9^ A natureza progressiva da doença e os seus efeitos a longo prazo sobre a função cardíaca e extracardíaca alertam para o momento apropriado de intervenção. No TRIGISTRY, um grande registro internacional multicêntrico, a taxa de sobrevida em 2 anos de 2.413 pacientes com RT funcional isolada grave foi estratificada de acordo com o TRI-SCORE, com base em oito parâmetros clínicos, biológicos e ecocardiográficos (idade, classe funcional pela NYHA, sinais de insuficiência cardíaca direita, dose diária de furosemida, taxa de filtração glomerular, nível de bilirrubina total, fração de ejeção ventricular esquerda e função ventricular direita).^10^ A intervenção cirúrgica ou transcateter bem-sucedida na válvula tricúspide foi associada a uma melhor sobrevida quando comparadas ao manejo conservador nas categorias de risco TRI-SCORE baixo e, em menor grau, intermediário, enquanto a sobrevida foi semelhante independentemente do tratamento na categoria de risco alto.^2^ Nosso caso ilustra a indicação de SVTT em uma paciente com TRI-SCORE intermediário, com sintomas refratários e função biventricular preservada, situação em que o referenciamento precoce possibilita um prognóstico favorável.

A SVTT ortotópica é uma alternativa promissora de tratamento devido à previsibilidade na redução da RT e da técnica de implante. No entanto, as limitações anatômicas ainda representam importante causa de exclusão de potenciais candidatos. A melhoria contínua dos dispositivos atuais e novas tecnologias, como LuX-Valve Plus, têm o potencial de aumentar as opções de tratamento e simplificar os procedimentos.
