# Supplementary material for: Primeira Realização de Implante Transcateter de Valva Tricúspide Via Transjugular com o Sistema Lux-Valve Plus Na América Latina. Um Relato de Caso
Source: Arq Bras Cardiol. 2024 Oct 8;121(10):e20240201. [Article in Portuguese] doi: 10.36660/abc.20240201 (PMC11634202; doi:10.36660/abc.20240201)
Supplement: Translation

# Translation: First Performance of Transjugular Transcatheter Tricuspid Valve Replacement with the Lux-Valve Plus System in Latin America. A Case Report

**DOI:** 10.36660/abc.20240201i

**Authors:** Vinicius Esteves, Pedro Beraldo de Andrade, Sergio Kreimer, Fernanda Almeida Esteves, Francisco Monteiro de Almeida Magalhães, Thomas Modine

**Affiliations:** 1 São Paulo SP Brazil Rede D'Or Sao Luiz, São Paulo, SP – Brazil; 2 Marília SP Brazil Santa Casa de Misericórdia de Marília – Cardiologia Invasiva, Marília, SP – Brazil; 3 Santo André SP Brazil Hospital e Maternidade Brasil, Santo André, SP – Brazil; 4 São Paulo SP Brazil Instituto do Coração do Hospital das Clínicas da Faculdade de Medicina da Universidade de São Paulo, São Paulo, SP – Brazil; 5 Bordeaux France Centre Hospitalier Universitaire de Bordeaux, Bordeaux, Nouvelle-Aquitaine – France

**Keywords:** Tricuspid Valve Insufficiency, Heart Failure, Heart Valve Prosthesis Implantation

## Introduction

The prevalence of tricuspid regurgitation (TR) increases with age, concomitant left-side heart disease, and chronic atrial fibrillation.^1^ Moderate or severe TR is associated with excess mortality and poor outcomes.^2^ The interventional treatment of choice is tricuspid valve repair, with a prosthetic ring capable of reducing the tricuspid annulus diameter, improving valve leaflet coaptation, and correcting regurgitation. Valve replacement is reserved for patients who do not have anatomical conditions for repair. However, an isolated surgical approach to the tricuspid valve continues to be rarely indicated, and there are still no data showing improved survival with TR surgical treatment alone, making this condition undertreated in clinical practice, despite the increased mortality rate in patients with moderate to severe TR clinically-only managed.^3^

Based on these observations, the treatment of TR has recently been shifting from a conservative to a more interventional approach. This shift has led to first-in-human attempts at transcatheter tricuspid valve interventions (TTVI). Several devices have been developed, with strategies based on reducing the valve annulus, improving coaptation between the leaflets, or valve replacement.^4^ In a propensity-matched case-control study, TTVI was associated with 40% greater survival and freedom from heart failure rehospitalization.^5^

The LuX-Valve (Jenscare Biotechnology Co., Ningbo, China) is a radial force–independent orthotopic transcatheter tricuspid valve replacement (TTVR) device whose feasibility and efficacy have been previously reported.^6,7^ The LuX-Valve Plus system is the second-generation version of the device and can be implanted through the jugular vein.^8^ We present the first performance of transjugular TTVR with the LuX-Valve Plus system in Latin America in a patient with symptomatic TR at high surgical risk.

## Case Report

A 78-year-old female patient with systemic arterial hypertension, atrial fibrillation, diabetes mellitus, dyslipidemia, peripheral arterial obstructive disease, and rheumatoid arthritis presented with signs and symptoms of chronic right heart failure, including peripheral edema, ascites, exercise intolerance, dyspnea, and poor functional capacity. Despite optimized clinical therapy, the patient has had recurrent hospital admissions due to worsening symptoms. Echocardiogram revealed preserved biventricular function, no signs of pulmonary arterial hypertension, significant biatrial enlargement (right atrial indexed volume = 50mL/m^2^ and left atrial indexed volume = 75mL/m^2^), severe (3+) TR, with effective regurgitant orifice (ERO) area = 0.4cm^2^, regurgitant volume = 42mL/beat, and tricuspid annular diameter = 46mm. Based on advanced age, 8.4% 30-day mortality risk by Society of Thoracic Surgeons (STS) score, and on a favorable computed tomography scan anatomical analysis, the Heart Team opted for TTVR with LuX-Valve Plus system 30-50.

LuX-Valve Plus system consists of four components: 1) a trileaflet prosthetic valve with bovine pericardium; 2) a self-expandable nitinol valve stent consisting of an atrial disc; 3) one interventricular septal anchor "tongue"; and 4) two expanded polytetrafluoroethylene-covered graspers (Figure 1). The procedure was performed under general anesthesia with transoesophageal echocardiographic and fluoroscopic guidance. The right internal jugular vein was punctured under ultrasound guidance, a 30 Fr introducer sheath was then placed into the vein, and subsequently, the delivery system was advanced. The valve was released, and the anterior leaflet-grasping clips were expanded. The delivery system was then withdrawn, allowing the capture of the anterior leaflet by the clips. The atrial disc was subsequently deployed, and the valve started to work. The septal tongue (anchoring component) was then deployed and immobilized by firing a 3-pronged nitinol anchor onto the ventricular septum. Finally, the valve was completely released, and the delivery catheter was drawn back and removed (Figure 2). Hemostasis was achieved with 2 Perclose ProGlide^™^ and a pre-closed technique.

**Figure 1:** LuX-Valve Plus System.

**Figure 2:** LuX-Valve Plus device implantation technique. A) Transjugular percutaneous access. B) System alignment and coaxialization. C) Ventricular release. D) Final release.

The discharge echocardiography showed significant TR reduction and less than mild TR at 30 days. The patient reported significant improvement in New York Heart Association (NYHA) functional status at 30-day follow-up.

## Discussion

The LuX-Valve Plus system is a transjugular TTVR system implanted through the jugular vein. The delivery system has the functions of bending, rotation, and expansion, which allow it to meet the requirements of valve positioning and ensure that the valve can be roughly coaxial with the autologous tricuspid valve. Twelve-month results of the first-generation LuX-Valve system implantation in six patients, through a small incision of the right chest and right atrium, confirmed its safety and efficacy, with significant improvements in mean transvalvular gradient, right heart sizes, conventional right ventricle function indices, and global longitudinal strain, reduction in NYHA functional class, and no significant paravalvular leakage in all patients but one, who died three months postoperatively due to moderate paravalvular TR and non-improvement of right heart failure.^7^

The first-in-human experience with this second-generation device demonstrated procedural success in all ten cases, with no intraprocedural mortality or conversion to open surgery. Unlike our case, in which the access site was fully percutaneous, the procedures were performed through a 3-4 cm cut in the skin of the right side of the neck and exposure of the right jugular. All patients reported NYHA functional status improvement and less than mild TR at 30-day echocardiographic control.^8^ Patients with severe pulmonary arterial hypertension (systolic pulmonary artery pressure ≥55 mmHg), other valve lesions requiring operative intervention, left ventricular ejection fraction <50%, congenital Ebstein's anomaly or arrhythmogenic right ventricular dysplasia, and untreated severe coronary artery disease were excluded.

The most used transcatheter intervention for patients with right heart failure is tricuspid transcatheter edge-to-edge repair. However, many patients are rendered unsuitable for the technique, as they present with large coaptation gaps, leaflet tethering, and torrential TR. For these patients, TTVR has emerged as an attractive alternative despite a real-world report of TTVR screening demonstrating a high screening failure rate, mainly related to large tricuspid annular diameter.^9^ The progressive nature of the disease and its long-term effects on cardiac and extra-cardiac function prompt attention to the appropriate time of intervention. In the TRIGISTRY, a large multicentre international registry, the 2-year survival rate of 2,413 patients with severe isolated functional TR was stratified according to TRI-SCORE, based on eight clinical, biological, and echocardiographic parameters (age, NYHA functional class, right-sided heart failure signs, daily dose of furosemide, glomerular filtration rate, total bilirubin level, left ventricular ejection fraction, and right ventricular function).^10^ Surgical or successful transcatheter intervention on the tricuspid valve was associated with better survival than conservative management in the low and, to a lesser extent, intermediate TRI-SCORE risk categories, while survival was similar irrespective of treatment in the high TRI-SCORE risk category.^2^ Our case illustrates the indication of TTVI in a patient with an intermediate TRI-SCORE, with refractory symptoms and preserved biventricular function, a situation in which early referral would envisage a favorable prognosis.

Orthotopic TTVR is a promising alternative to repair due to the predictability of TR reduction and implant technique. However, anatomical limitations exclude many potential candidates. Continuous improvement of current devices and new technologies like LuX-Valve Plus have the potential to increase treatment options and simplify procedures.
